# Supplementary material for: Health Risk Assessment of Inhalation Exposure to Airborne Particle-Bound Nitrated Polycyclic Aromatic Hydrocarbons in Urban and Suburban Areas of South China
Source: Int J Environ Res Public Health. 2022 Nov 23;19(23):15536. doi: 10.3390/ijerph192315536 (PMC9739065; doi:10.3390/ijerph192315536)
Supplement: Supplementary file 1 [file ijerph-19-15536-s001.zip › ijerph-1976304-supplementary.pdf]

International Journal of
Environmental Research and Public Health

MDPI

Supplementary Material

Health Risk Assessment of Inhalation Exposure to Airborne Particle–bound Nitrated Polycyclic Aromatic Hydrocarbons in Urban and Suburban Areas of South China

Peng Gao [1], Feng Deng [1], Weishan Chen [1], Yijia Zhong [1], Xiaolu Cai [1], Wenmin Ma [2], Jian Hu [3,*] and Shuran Feng [4,*]

[1] Guangzhou Panyu Polytechnic, Guangzhou, PR China
[2] Tianjin Key Laboratory of Water Resources and Environment, School of Geographic and Environmental Sciences, Tianjin Normal University, Tianjin, PR China
[3] Research Center for Eco-Environmental Sciences, Chinese Academy of Sciences, Beijing, PR China
[4] School of Business, Hong Kong Baptist University, Hongkong, PR China
* Correspondence: jianhu@rcees.ac.cn (J.H.); woshisophie0066@163.com (S.F.)

2. Materials and Methods

2.2. Sample analysis

The analysis of nitro-PAHs and PAHs required two instrumental runs. Total 3 nitro-PAHs were detected using an Agilent 7000A GC/MS Triple Quadrupole System (Agilent Technologies Inc, USA) and quantitatively analyzed in MRM mode. Ion source: inert ion source (EI). Chromatographic column: Agilent 19091J-433 HP-5 (30 m x 0.25mm x 0.25 mm). The injected volume was 1 μL in splitless mode. The parameters were as follows: inlet temperatures were 240 ◦C for nitro-PAHs, 80 ◦C ramp to 240 ◦C (at a rate of 20 ◦C / min) and held for 7.5 min, then ramp to 300 ◦C (at a rate of 30 ◦C /min) and held for 2.5 min; the initial temperature of GC was 80 ◦C (held 3 min) for PAHs (Naphthalene-d^8 and phenanthrene-d^{10}), and then heat to 200 ◦C (at a rate of 10 ◦C /min) and held for 4 min, last heat to 300 ◦C (at a rate of 6 ◦C /min) and held for 8 min. Due to various isomers were contained in detected nitro-PAHs and PAHs, different oven temperature program were tried to select the optimal method, which has not only the better analytical peaks but also the shorter separation time [1]. The initial collision energy (CE) was set as 10–60 eV to determine the characteristic fragment ions. Lastly, CE was further optimized with the increase in sensitivity under Multi Reaction Monitor (MRM) mode. Different CE parameters were selected from 5 eV to 60 e V and the other parameters were same, appropriate CE parameter was determined by observing the corresponding signal conditions of each tar-get peak for 3 nitro-PAHs [1].

3 nitro-PAHs were separated and the analysis time was 26.397 min for 2-Nitrofluorene (CE:15 eV), 27.170 min for 9-Nitroanthracene (CE:15 eV) and 32.241 min for 1-Nitropyrene (CE:25 eV).

Table S1. TEF data of the objective nitro-PAH.

Compound	TEF	Compound	TEF
9-nitroanthracene (9-NT)	0.0032	2-nitrofluorene (2-NF)	0.01
1-nitropyrene (1-NP)	0.03		

Table S2. Parameters used in the average daily exposure assessment.

	Unit	Value	References
C (Nitro-PAHs concentrations in $PM_{2.5}$ and TSP)	ng/m^3		In this study
Inhalation rate (IR)	m^3/day	Male: 15.2<br>Female: 11.3<br>Children: 8.7	[2]
F	h/24h	Outdoor: 3/24<br>Indoor: 21/24	[2]
Exposure frequency (EF)	day/year	Summer: 90<br>Winter: 90	[2]
Exposure duration (ED)	year	Male/Female: 30<br>Children: 10	[2]
Body weight (BW)[a]	kg	Male: 70<br>Female: 60<br>Children: 36	[2]
Averaging life span (AT)	day	70 x 365	[2]

Table S3. Correlation coefficients and p-values between total nitro-PAHs and meteorological parameters with different airborne particle in Haizhu District in 2019.

**Nitro-PAHs**	**SO_2**	**CO**	**NO_2**	**O_3**	**Temperature**	**Solar radiation**	**Wind speed**
Winter in 2019							
$PM_{2.5}$	0.171	0.302	0.404*	0.441*	0.431*	0.561*	0.511*
TSP	0.262	0.263	0.471*	0.383*	0.503*	0.524*	0.470*
Summer in 2019							
$PM_{2.5}$	-0.344	0.21	0.450*	0.531*	-0.270	0.550*	-0.442*
TSP	0.224	0.33	0.502*	0.410*	-0.310	0.492*	-0.381

* means correlation is significant at $p < 0.05$.

References

1. Zhang Z.F.; Chen J.C.; Zhao Y.X.; Wang L.; Teng Y.Q.; Cai M.H.; Zhao Y.H.; Nikolaev A.; Li Y.F. Determination of 123 polycyclic aromatic hydrocarbons and their derivatives in atmospheric samples. Chemosphere 2022, 296, 134025.
2. USEPA (United States Environmental Protection Agency), Risk Assessment Guidance for Superfund. Volume I: Human Health Evaluation Manual. 2004.
